# Artificial Selection for Whole Animal Low Intrinsic Aerobic Capacity Co-Segregates with Hypoxia-Induced Cardiac Pump Failure

**DOI:** 10.1371/journal.pone.0006117

**Published:** 2009-07-01

**Authors:** Nathan J. Palpant, Michael L. Szatkowski, Wang Wang, DeWayne Townsend, Fikru B. Bedada, Lauren G. Koch, Steven L. Britton, Joseph M. Metzger

**Affiliations:** 1 Department of Molecular and Integrative Physiology, University of Michigan Medical School, Ann Arbor, Michigan, United States of America; 2 Division of Neonatology, Drexel University College of Medicine, St. Christopher's Hospital for Children, Philadelphia, Pennsylvania, United States of America; 3 Department of Integrative Biology and Physiology, University of Minnesota Medical School, Minneapolis, Minnesota, United States of America; 4 Department of Physical Medicine and Rehabilitation, University of Michigan Medical School, Ann Arbor, Michigan, United States of America; University of Las Palmas de Gran Canaria, Spain

## Abstract

Oxygen metabolism is a strong predictor of the general health and fitness of an organism. In this study, we hypothesized that a divergence in intrinsic aerobic fitness would co-segregate with susceptibility for cardiovascular dysfunction. To test this hypothesis, cardiac function was assessed in rats specifically selected over nineteen generations for their low (LCR) and high (HCR) intrinsic aerobic running capacity. As an integrative marker of native aerobic capacity, run time to exhaustion between LCR and HCR rats had markedly diverged by 436% at generation nineteen of artificial selection. *In vivo* assessment of baseline cardiac function by echocardiography and catheter-based conductance micromanometry showed no marked difference in cardiac performance. However, when challenged by exposure to acute hypoxia, cardiac pump failure occurred significantly earlier in LCR rats compared to HCR animals. Acute cardiac decompensation in LCR rats was exclusively due to the development of intractable irregular ventricular contractions. Analysis of isolated cardiac myocytes showed significantly slower sarcomeric relaxation and delayed kinetics of calcium cycling in LCR myocytes compared to HCR myocytes. This study also revealed that artificial selection for low native aerobic capacity is a novel pathologic stimulus that results in myosin heavy chain isoform switching in the heart as shown by increased levels of β-MHC in LCR rats. Together, these results provide evidence that alterations in sub-cellular calcium handling and MHC isoform composition are associated with susceptibility to compensatory cardiac remodeling and hypoxia induced pump failure in animals with low intrinsic aerobic capacity.

## Introduction

The prevalence of metabolic syndrome has come to be regarded as a window into the general health of a population [1]. Aspects of metabolic syndrome associated with cardiovascular disease include abdominal obesity, atherogenic dyslipidemia, elevated blood pressure, insulin resistance with or without glucose intolerance, a pro-inflammatory state, and a pro-thrombotic state [2]. Studies in the U.S. have shown that nearly a quarter of the population (an estimated 47 million people) have metabolic syndrome [3].

Previous studies of mono and dizygotic twins have shown that about 70% of the variation in aerobic capacity is due to inheritance [4]. Furthermore, cross-sectional and prospective studies have found that levels of physical activity and fitness are inversely related to the prevalence of metabolic syndrome [5]. These and other studies suggest a link between intrinsic aerobic capacity, based on genetic and environmental influences, and complex disease states [5], [6], [7], [8], [9].

Clinically relevant research models for metabolic syndrome must take into account the interaction between individual polygenic traits and environmental influences. In recent years, one such model has emerged providing a means to scientifically dissect complicated interactions at all levels of biological organization. As illustrated in previous studies, we have generated a unique breeding scheme based on inherent aerobic running capacity that has resulted in a significant divergence in native running capacity between two populations, termed low capacity runners (LCR) and high capacity runners (HCR) [10], [11], [12].

Previous reports on these rats have shown a significant divergence in risk factors associated with metabolic syndrome that align with selection for low versus high intrinsic aerobic exercise capacity. More specifically, risk factors associated with cardiovascular dysfunction have markedly diverged with aerobic capacity including cardiac energy substrate utilization [13], expression of key mitochondrial proteins [12], oxygen transport [14], [15], [16], and susceptibility to cardiac arrhythmias [17].

In light of our current level of knowledge about this animal model, several important questions remain. First, catheter-based conductance micromanometric analysis of cardiac hemodynamics has not been performed on this animal model. This methodology provides access to real time pressure and volume data elucidating cardiac pump performance of LCR and HCR rats at baseline, during catecholaminergic manipulation, and in response to an acute hypoxic stress. Furthermore, *in vitro* analysis of myocyte calcium handling, contractile kinetics, and biochemical analysis of protein post-translational modifications provide a sub-cellular perspective into a molecular and cellular basis for pathologies observed *in vivo*. Specific markers of heart failure such as activation of the fetal gene program (e.g. MHC isoform switching) and correlative alterations in thyroid hormone function also have not been assessed in this model of metabolic syndrome. We hypothesized that data derived from these cell and molecular assays would provide important mechanistic insights into the underlying cardiovascular pathologies observed within this disease model.

Rats used in this study were derived from 19 generations of selection correlating with a 436% divergence in intrinsic aerobic running capacity. Based on this divergence in inherent aerobic fitness and previous evidence of diverging susceptibilities for cardiovascular risk factors between LCR and HCR rats [12], [17], [18], [19], we hypothesized that indexes of cardiac function would co-segregate with intrinsic aerobic capacity. This focused analysis on the heart provides insights into the complex pathophysiology associated with metabolic syndrome and supports the hypothesis that low intrinsic aerobic capacity portends an increased susceptibility to cardiac dysfunction during cardiac stresses such as oxygen deprivation.

## Methods

### Animal model

Adult rats selectively bred for their inherent high capacity (HCR) or low capacity (LCR) endurance treadmill running, as previously outlined [12], were used for this study. All animals were given food and water ad libitum. The procedures used in this study are in agreement with the guidelines of the University of Michigan and approved by the University of Michigan Committee on the Use and Care of Animals.

### Conductance Micromanometry

Real-time measurements of *in vivo* cardiovascular hemodynamics were obtained using conductance micromanometry as previously performed [20]. Rats were anesthetized and ventilated via a tracheal cannulation and ventilated via a pressure controlled ventilator with 1% isoflurane at a peak inspiratory pressure of 15 cm H_2_O and a respiratory rate of 60 breaths/min. An arteriotomy was then performed on the right carotid artery followed by insertion of a 2.0 French Millar pressure-volume catheter (SPR-838; Millar Instruments Inc., Houston, Texas, USA). The catheter was subsequently advanced into the junction of the carotid artery and aorta at which point arterial pressure analysis was performed. The catheter was then advanced further into the left ventricle for cardiac hemodynamic analysis. Pressure-volume loops were collected on line at 1 kHz. Data were analyzed with Biobench and PVAN software. Pancuronium bromide was administered IP to block spontaneous respiratory muscle activity (0.4 mg/kg). After obtaining baseline hemodynamics (ventilated with isoflurane and O_2_), rats received a continuous infusion of esmolol (250 ug/kg/min) for five minutes. After returning to baseline hemodynamics, rats were then infused with dobutamine (42 ug/kg/min) for two minutes. After returning to baseline, rats were subjected to a long term hypoxia challenge (ventilation with 7% oxygen balanced with nitrogen). For each experimental animal, cardiac function was assessed for all manipulations described above. All hemodynamic values reported in this study were derived during ventilation with 100% oxygen (baseline, dobutamine, and esmolol) or during exposure to 7% oxygen (hypoxia). Isoflurane is known to have a cardioprotective effect which may have attenuated the severity of the hypoxia challenge on these animals [21]. Even so, equal exposure to animals in this study makes comparative analysis of cardiac performance during this challenge informative to our understanding of energy metabolism and cardiovascular function in the context of metabolic disease.

Using the LV pressure waveform as a marker of cardiac contraction, data were acquired during hypoxia until either sustained irregular ventricular contractions or acute systolic pump failure occurred. Irregular contractions were defined by calculating R–R intervals. Irregular contractile events were defined as positive or negative deflections of at least 15 msec away from the baseline intervals. Baseline was defined as the mean value over 50 beats before or after a given period excluding those beats identified as irregular, as follows. Close scrutiny of R–R intervals revealed patterns of intractable irregular contractions used to demarcate termination of the experiment in certain animals. These criteria include: greater than 2 R–R intervals of 200 ms or more above baseline within 20 beats, greater than 15 R–R intervals of 25 ms or more above baseline within 20 beats, and greater than 30 R–R intervals of 120 ms or more above baseline within 100 beats. Acute cardiac failure due to pump dysfunction was defined as LV pressure reaching 60% of initial peak systolic pressure. Whether acute pump failure was determined to be caused by irregular ventricular contractions or pump dysfunction, rats were immediately recovered using 100% O_2_ in order to obtain measurements for instrument calibration. All pressure waveform data was used to assess irregular beat intervals using programs and macros derived from LabVIEW and Microsoft Excel.

### Echocardiography

Anesthesia was induced with 3% isoflurane and then maintained at 1% for the duration of the procedure. Transthoracic echocardiography was performed in the supine or left lateral position. Two-dimensional, M-mode, Doppler and tissue Doppler echocardiographic images were recorded using a Visual Sonics' Vevo 770 high resolution *in vivo* micro-imaging system. We measured systolic and diastolic dimensions and wall thickness in M-mode in the parasternal short axis view at the level of the papillary muscles. Fractional shortening and ejection fraction were calculated from the M-mode parasternal short axis view. We assessed diastolic function by conventional pulsed-wave spectral Doppler analysis of mitral valve inflow patterns (early [E] and late [A] filling waves). Doppler tissue imaging (DTI) was used to measure the early (E_a_) and late (A_a_) diastolic tissue velocities of the lateral annulus. Rats were assessed for baseline cardiac function while being ventilated with 100% oxygen.

### Ventricular myocyte isolation and primary culture

LCR and HCR rats were anaesthetized by inhalation of isoflurane followed by i.p. injection of heparin (1500 U/kg) and Nembutal (162.5 U/kg). Following enzymatic digestion by retro-grade perfusion with collagenase and hyaluronidase and gentle mincing of the cardiac ventricles, cardiac myocytes were plated on laminin-coated glass coverslips (2×10^4^ myocytes/coverslip) and cultured in M199 media (Sigma, supplemented with 10 mmol/L glutathione, 26.2 mmol/L sodium bicarbonate, 0.02% bovine serum albumin, and 50 U/ml penicillin-streptomycin, with pH adjusted to 7.4, as described previously [22]).

### Contractility and calcium measurements in single myocytes

Myocytes were loaded with fura-2AM (a ratiometric calcium indicator, 2 µmol/L) for 10 minutes at room temperature. Following a de-esterification period of 20 min in M199 media (1.8 mmol/L Ca2+), loaded cells were placed on the inverted microscope (Nikon, Eclipse TE2000) and stimulated at 0.2 Hz. The chamber's temperature was maintained at 37°C. Fura-2 fluorescence was measured using an IonOptix spectrophotometer (Stepper Switch). Initially, fura was excited by 360 nm (the Fura isosbestic point, a calcium independent measure of fura fluorescence) light and then continuously with 380 nm (calcium dependent measure of fura fluorescence) light. Emitted fura fluorescence was collected by the 40× objective, passed through a 510 nm filter and detected by a photomultiplier tube. Ratiometric data was collected and analyzed online using commercial software (IonOptix Corp.). Together with calcium measurements, cell contractility was also measured. Myocyte images were collected (240 Hz) using a CCD camera (MyoCam, IonOptix). Myocytes that did not follow the pacing protocol (0.2 Hz) were excluded, as were myocytes with a resting sarcomere length less than 1.75 µm. Velocity of shortening was measured in these electrically paced myocytes. Velocity of shortening was calculated using IonOptix software, which calculates the maximum of the first derivative of the contraction transient during the shortening phase. Likewise, the velocity of re-lengthening was measured as the maximum of the first derivative of the re-lengthening phase.

### Immunoblot detection

Hearts were isolated, snap frozen in liquid nitrogen, homogenized and placed in Laemmli sample buffer. Proteins were separated by SDS-PAGE and transferred to a nitrocellulose membrane for immunodetection. After blocking in 5% milk (in Tris-buffered saline), membranes were probed with specific antibodies directed against β-myosin heavy chain (ATCC), troponin I phosphorylation at serine 23/24 (Cell Signaling Technology), SERCA2a (Chemicon), phospholamban (Upstate), phospho-phospholamban (Upstate), calsequestrin (ABR), and the sarcolemmal sodium-calcium exchanger (Swant). Protein detection for actin was used for loading control. Indirect immunodetection was carried out using a fluorescently labeled secondary antibody (Rockland, IRDye 680 conjugated affinity purified; 1∶5000). Western blot analysis was accomplished using the infrared imaging system, Odyssey (Li-Cor, Inc.) and images analyzed using Odyssey software v. 1.2.

### Thyroid hormone analysis

Total serum was extracted from mice immediately after decapitation. Samples were submitted for thyroid hormone panel analysis at the University of Michigan hospital chemistry lab.

### Quantitative RT-PCR

Total RNA was extracted from ventricles of LCR and HCR rats using Trizol reagent (Invitrogen) according to the manufacture's instructions. Quality of total RNA was visualized via electrophoresis on a 1.0% agarose gel. cDNA was generated from 1 µg RNA by reverse transcriptase reaction using TaqMan RT reagent kit (Applied Biosystems, Foster City, CA). All RT reactions to generate cDNA were performed on the MJ Research PTC-200 thermocycler (Global Medical Instrumentation, Inc). Real-time quantitative PCR was performed on cDNAs using the 2x SYBR Green universal PCR master mix Kit (Applied Biosystems, Foster City, CA) and run on Realplex^2^ master cycler epgradient (Eppendorf). All qPCR assays were performed in duplicate on 12 LCR and 12 HCR rats and normalized against wild type rat. Cycling conditions consisted of one cycle of 95°C for 10 min, and then 40 cycles of 95°C for 15 s followed by 60°C for 1 min. The representative normalized average expression values from independent experiments are shown for each gene.

Relative quantitative analysis of gene expression was conducted according to the 2^−ΔΔCT^ method. 18s rRNA was used as endogenous internal standards for gene expression analysis to determine the abundance of amplified target gene within the same sample. Primers were designed using Primer select (DNAstar lasergene 7). The amplification efficiency for all primers was in the range of 90 to 100% as determined by standard curve. All primers displayed single melting curve.

The following primers were used for RT-PCR:

Rat ANF-foward- TGAAAAGCAAACTGAGGGCT

Rat ANF-reverse- CTCCAGGAGGGTATTCACCA

Rat BNP- foward - CAGCTCTCAAAGGACCAAGG

Rat BNP- reverse AGAGCTGGGGAAAGAAGAGC

Rat αSKactin- foward - GCATGCAGAAGGAGATCACA

Rat αSKactin- reverse - CATAGCACGATGGTCGATTG

Rat βMHC- foward - AGATCGAGGACCTGATGGTG

Rat βMHC- reverse - GATGCTCTTCCCAGTTGAGC

Rat 18s rRNA- foward -TTTGTTGGTTTTCGGAACTGAGGC

Rat 18s rRNA- reverse -GGCATCGTTTATGGTCGGAACTACG

### Statistics

All results are expressed as mean±SEM. All two-group comparisons were assessed by two-tailed *t*-test. All multi-group comparisons were assessed using two way analysis of variance (ANOVA) with Tukey post-hoc test. Survival during acute hypoxia was assessed by the Fisher exact test.

## Results

### Analysis of intrinsic aerobic running capacity

**Figure 1:**
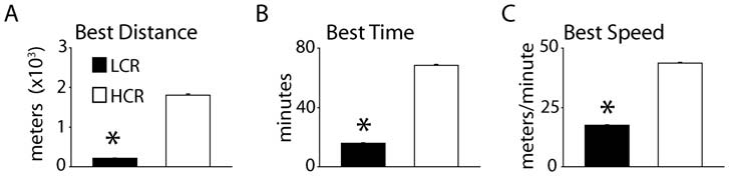
Intrinsic aerobic running capacity of LCR and HCR rats. Mean values derived from a treadmill run assessing aerobic running capacity showing best distance run (a), best time (b), and best running speed (c) (n = 14/group). Values are expressed as mean±SEM. *P<0.05. HCR, high capacity runner, LCR, low capacity runner.

Determination of intrinsic (non-trained) aerobic running capacity was derived by a treadmill exercise test [12]. The single best daily run of five trials for each rat was considered the trial most closely associated with the animal's heritable component of exercise endurance. The founder population (generation 0) had a capacity to run for 355±144 m (23.1 min) until exhausted. Previous studies have reported that the treadmill-running capacity for LCR rats decreased 16 m per generation and increased in HCR rats by 41 m per generation in response to artificial selection [12]. At generation 11, the LCRs averaged 191±70 m (run time: 14.3 min), and the HCRs ran for 853±315 m (run time: 41.6 min). By comparison, the current study used rats selected for 19 generations which showed further divergence in native aerobic running capacity between LCR and HCR rats. Specifically, there were significant differences revealed in measures of best distance run ([LCR] 217.0±6.3 vs. [HCR] 1813.7±30.7 meters, P<0.05), best time ([LCR] 16.0±0.4 vs. [HCR] 68.2±0.7 minutes, P<0.05), and best speed ([LCR] 17.6±0.2 vs. [HCR] 43.6±0.4 meters/minute, P<0.05) (Figure 1a–c). These differences in intrinsic aerobic running performance prompted further investigation into possible underlying variations in cardiovascular function between LCR and HCR rats.

### Cardiac function assessed by echocardiography

**Figure 2:**
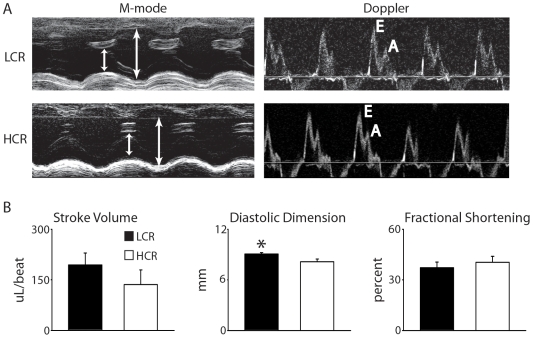
Analysis of baseline cardiovascular function by echocardiography. (a) Echocardiographic images showing systolic and diastolic dimensions (arrows) and wall thickness measured by M-mode in the parasternal short axis view at the level of the papillary muscles (left) as well as diastolic function assessed by conventional pulsed-wave spectral Doppler analysis of mitral valve inflow patterns (early [E] and late [A] filling waves) (right). (b) Summarized mean echo data of stroke volume, diastolic dimension, and fractional shortening. Values are expressed as mean±SEM. *P<0.05. HCR, high capacity runner (n = 6); LCR, low capacity runner (n = 5).

Assessment of baseline cardiac systolic and diastolic function as well as cardiac LV geometry was performed by echocardiography. Overall, these data showed minor differences in heart performance under resting conditions between LCR and HCR rats. Most measures of cardiovascular systolic and diastolic function and LV geometry were not different between groups (e.g. fractional shortening (FS), cardiac output (CO), isovolumic relaxation time (IVRT), and LV wall thickness) (Figure 2a and b, Table 1). Left ventricular geometrical measures, however, showed a significantly larger ventricular diastolic dimension in LCR rats compared to HCR rats (LVDd [LCR] 9.1±0.2 vs. [HCR] 8.2±0.3 mm, P<0.05).

**Table 1:** Baseline Function by Echocardiography.

Parameter	HCR	LCR
HR (bpm)	359.3±15.7	323.5±16.9
PW d (mm)	1.6±0.1	1.7±0.0
MV E (mm/s)	1270.8±96.4	1142.3±113.4
E/A	1.1±0.1	1.1±0.1
IVRT (ms)	20.6±1.9	22.5±3.3
TEI Index	0.35±0.0	0.36±0.0
E/Ela	37.8±4.7	35.4±9.2
LVDs (mm)	4.9±0.5	5.7±0.4

Echocardiographic parameters: heart rate (HR), LV posterior wall at diastole (PW d), fractional shortening (FS), flow velocity of the mitral valve E wave (MV E), flow velocities of the mitral valve E and A waves expressed as a ratio (E/A), isovolumic relaxation time (IVRT), TEI index, the flow velocity of the mitral valve E wave to the tissue velocity of the lateral annular E wave ratio (E/Ela), and LV dimension during diastole (LVDd) and systole (LVDs). All values are expressed as mean±s.e.m.

* P<0.05.

HCR, high capacity runner (n = 6), LCR, low capacity runner (n = 5).

Gross heart and body weight analysis revealed that LCR rats had significantly larger hearts by mass correlating with a significantly larger body weight (Table 2). However, when normalized for body weight, LCR rats had a significantly smaller heart size compared to HCR rats (HW/BW ratio [LCR] 2.6±0.2 (×10^−3^) vs. [HCR] 3.1±0.1 (×10^−3^), P<0.05).

**Table 2:** Heart and Body Weight Analysis.

	HCR	LCR
Heart Weight (g)	1.27±0.0	1.46±0.1*
Body Weight (g)	416.8±14.5	570.3±23.7*
HW/BW ratio (×10^−3^)	3.1±0.1	2.6±0.2*

All values are expressed as mean±s.e.m.

* P<0.05.

HCR, high capacity runner (n = 11); LCR, low capacity runner (n = 12).

### Cardiac function assessed by real-time conductance micromanometry

**Figure 3:**
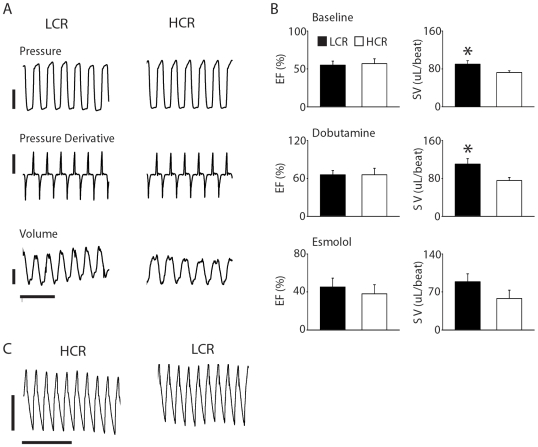
(a) Representative raw traces derived by conductance micromanometry of LV pressure (top; bar = 60 mmHg), derivatives (middle; bar = 10,000 mmHg/sec), and volume (bottom; bar = 50 uL) at baseline in LCR and HCR rats (time scale = 0.4 sec). (b) Summarized mean data for ejection fraction (EF) and stroke volume (SV) at baseline, during dobutamine infusion, and during esmolol infusion (n = 4–9/group for all hemodynamic data). (c) Raw traces of arterial blood pressure acquired by micromanometry measurements from the carotid artery and table of mean blood pressure values for LCR and HCR rats (pressure scale = 25 mmHg, time scale = 1 sec) (n = 9/group). Values are expressed as mean±SEM. *P<0.05. HCR, high capacity runner, LCR, low capacity runner.

To more precisely analyze cardiac pump performance *in vivo*, rats were instrumented with a Millar pressure-conductance catheter in the left ventricle. To measure the full range of cardiac function, LCR and HCR rats were also treated to maximally activate or inhibit beta adrenergic signaling. Measures of systolic function (e.g. ejection fraction), and diastolic function (e.g. end diastolic pressure (EDp), and LV pressure Tau) were not different between low and high capacity rats (Figure 3a and b and Table 3). Consistent with a higher end diastolic dimension as determined by echocardiography, there was increased stroke volume among low capacity animals at baseline ([LCR] 90.1±7.2 vs. [HCR] 72.4±3.7 uL, P<0.05).

**Table 3:** Real-time Hemodynamic Function.

Parameter	HCR	LCR
**Baseline**		
HR (bpm)	378.8±12.6	388.6±10.7
ESp (mmHg)	135.6±4.4	133.0±5.0
EDp (mmHg)	13.7±1.1	14.4±0.9
CO (uL/min)	28, 884±2,684	35,004±2,945
SV (uL/beat)	72.4±3.6	90.1±7.2*
+dP/dt (mmHg/s)	11,635±726	12,188±639
−dP/dt (mmHg/s)	−10,506±457	−10,649±870
Tau w (msec)	10.6±0.4	10.5±0.5
**Dobutamine**		
HR (bpm)	455.0±12.6	448.0±21.1
ESp (mmHg)	123.3±9.8	129.4±11.7
EDp (mmHg)	9.3±1.2	8.2±1.0
CO (uL/min)	38089.8±4867	49916.2±6546
SV (uL/beat)	75.4±6.9	110.2±11.6*
+dP/dt (mmHg/s)	20,106±1,065	18,726±1,758
−dP/dt (mmHg/s)	−10,576±1,233	−10,591±1,533
Tau w (msec)	8.4±0.4	7.6±0.3
**Esmolol**		
HR (bpm)	340.5±36.9	316.5±14.5
ESp (mmHg)	109.0±15.3	105.0±23.6
EDp (mmHg)	3.8±0.9	3.4±1.5
CO (uL/min)	19537.5±4949	28658.0±6099
SV (uL/beat)	58.1±15.1	88.9±14.6
+dP/dt (mmHg/s)	7,237±880	7,544±1,165
−dP/dt (mmHg/s)	−7,680±891	−8,092±1,805
Tau w (msec)	10.7±0.6	10.0±0.2
**Hypoxia**		
HR (bpm)	438.7±16.5	449.2±35.2
ESp (mmHg)	116.7±13.6	137.0±14.1
EDp (mmHg)	16.2±1.2	18.8±1.8
CO (uL/min)	32185.7±2005	53030.2±10051
SV (uL/beat)	73.1±6.3	118.0±20.5*
+dP/dt (mmHg/s)	11,772±3,060	14,571±1,714
−dP/dt (mmHg/s)	−7,913±1,716	−10,407±1,626
Tau w (msec)	12.7±1.7	10.0±0.4
**Arterial Pressure**		
Systolic (mmHg)	138.3±9.7	151.4±5.0
Diastolic (mmHg)	91.2±5.9	90.9±6.9
MAP (mmHg)	108.7±6.6	113.7±4.7

Parameters: heart rate (HR), end systolic pressure (ESp), end diastolic pressure (EDp), cardiac output (CO), stroke volume (SV), the positive and negative derivatives of pressure development (+dP/dt and −dP/dt, respectively), and LV pressure Tau (Tau w). Hypoxia values were derived at 5 minutes into the challenge. Arterial pressure values were derived by conductance catheterization in the right carotid artery. All values are expressed as mean±s.e.m.

* P<0.05.

HCR, high capacity runner (Baseline: n = 11, Dobutamine: n = 8, Esmolol: n = 4, Hypoxia: n = 7), LCR, low capacity runner (Baseline: n = 9, Dobutamine: n = 8, Esmolol: n = 4, Hypoxia: n = 5).

Next, during dobutamine infusion to maximally stimulate the adrenergic response in the heart, LCR and HCR rats showed similar inotropic responsiveness based on significant increases in the ejection fraction (EF) and maximal positive pressure derivative (+dP/dt) (Figure 3 and Table 3). However, during dobutamine infusion, LCR rats continued to maintain a significantly higher stroke volume compared to HCR rats ([LCR] 110.2±11.6 vs. [HCR] 75.4±6.9 uL, P<0.05). Other measures of systolic and diastolic function were not different during dobutamine infusion. During infusion of esmolol, for blockade of β-adrenergic signaling, no differences in cardiovascular performance were observed between LCR and HCR rats (Figure 3 and Table 3). Analysis of the delta response to dobutamine and esmolol as surrogate markers of cardiac reserve and adrenergic tone, respectively, showed no differences between groups (data not shown).

Systemic arterial pressures were analyzed by micromanometry in the right carotid artery. Based on analysis of systolic, diastolic and mean pressure measurements, these data showed that HCR and LCR rats had no statistically significant difference in mean arterial pressure ([LCR] 113.7±4.7 vs. [HCR] 108.7±6.6 mmHg) (Figure 3c and Table 3).

### Cardiac function assessed during acute exposure to hypoxia

**Figure 4:**
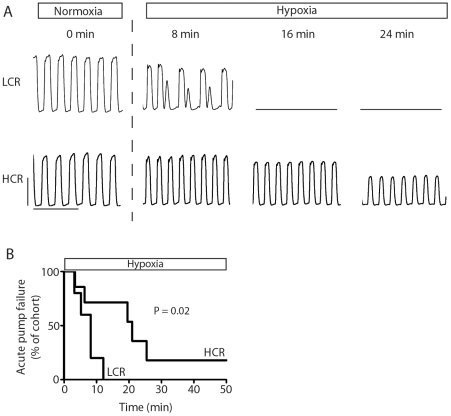
Analysis of cardiac function during acute exposure to hypoxia. (a) Representative raw traces of LV pressure at baseline and during the time course of exposure to hypoxia showing development of intractable irregular contractions in LCR rats and progressive diminution in pump function in HCR rats. (b) Survival graph showing cumulative mortality across a 50 minute acute hypoxic challenge. HCR, high capacity runner (n = 7); LCR, low capacity runner (n = 5).

The hypothesis was tested that differences in heritable oxygen carrying capacity [23] would result in diverging cardiac responses between LCR and HCR animals during an acute hypoxic challenge *in vivo*. To assess this, LCR and HCR rats were exposed to hypoxia (ventilation with 7% oxygen) and assessed for cardiac function and survival with real-time *in vivo* conductance micromanometry analysis (Figure 4, Table 3). Results revealed significant differences in cardiac responsiveness to hypoxic stress that segregated with intrinsic running capacity. During hypoxia, rats with low intrinsic exercise capacity (LCR) underwent acute cardiac pump failure more rapidly than rats with high intrinsic exercise capacity (HCR) (acute cardiac failure curve, P = 0.02) (Figure 4).

**Figure 5:**
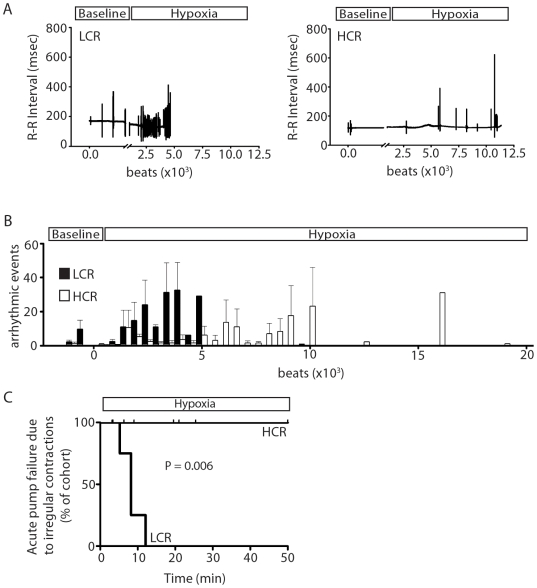
Analysis of cardiac irregular contractions during acute exposure to hypoxia. (a) Representative graphical presentations of fluctuations in the periodicity of the pressure waveform, defined as irregular ventricular contractions (see Methods) between LCR rats (left) and HCR rats (right) during the time course of exposure to hypoxia. (b) Average number of irregular contractions within a given segment of 500 beats among LCR and HCR rats at baseline and during hypoxia. 2 Way ANOVA analysis of the data showed a statistically higher number of irregular ventricular contractions in LCR vs. HCR rats (P<0.05). (c) Survival graph showing cumulative mortality due exclusively to intractable irregular contractions across a 50 minute acute hypoxic challenge. HCR, high capacity runner (n = 7); LCR, low capacity runner (n = 5).

The underlying etiology of cardiac pump failure was further analyzed to determine the mechanism of decompensation observed in HCR and LCR animals during this acute hypoxic challenge. Specifically, contractile failure was defined as systolic pump failure or the occurrence of defined patterns (see Methods) of intractable irregular ventricular contractions. Analysis of the cause of acute cardiac decompensation revealed that low capacity runners (LCR) experienced sustained irregular ventricular contractions without a marked diminution in LV pressure specifically attributable to systolic performance (Figure 4a and 5). Since electrocardiographic (ECG) analysis was not performed in parallel, no conclusions are made here regarding the specific etiology of these irregular beats based on the current data set. Analysis of the periodicity of ventricular pressure traces showed a significantly higher cumulative number of irregular beats in low capacity runners than high capacity runners during the time course of exposure to an acute hypoxic challenge (P<0.05) (Figure 5a and b). Analysis of survival based on cardiac decompensation due exclusively to fatal sustained irregular ventricular contractions accentuates the propensity for cardiac pump failure among LCR rats compared to HCR rats (P<0.05) (Figure 5c). Thus, HCR rats survived significantly longer during the hypoxic challenge. Cardiac pump failure of HCR rats was exclusively characterized as a diminution in cardiac systolic performance during hypoxia with no failures attributable to the development of fatal irregular contractions (Figure 5c).

### Functional analysis of LCR and HCR isolated cardiac myocytes

**Figure 6:**
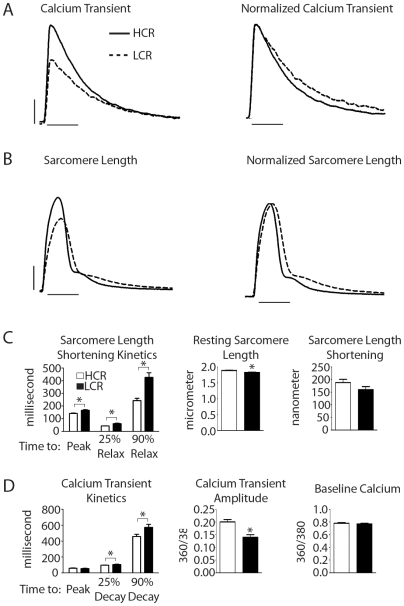
Calcium and contractility kinetics of acutely isolated myocytes. Representative raw traces of (a) calcium transients (vertical bar = 0.05 360/380 ratio, horizontal bar = 0.25 seconds) and (b) sarcomere length shortening kinetics (vertical bar = 0.05 um, horizontal bar = 0.25 seconds) from acutely isolated cardiac myocytes derived from LCR (dotted line) and HCR (solid line) rats. Raw transient traces (left) and normalized transients (right). (c) Mean summarized data showing sarcomere length shortening kinetics and (d) calcium transient kinetics from LCR and HCR rats (n = 45 myocytes/group). LCR, low capacity runner; HCR, high capacity runner. Values are expressed as mean±SEM. *P<0.05.

We hypothesized that the basis of LCR heart susceptibility to this hypoxic challenge resides in a functional contractile defect at the single myocyte level. To test this, cardiac myocytes were isolated from adult LCR and HCR rats and assessed for contractile properties. Calcium handling was also measured using the calcium indicator dye, fura-2AM. Interestingly, kinetics of the calcium transient revealed that LCR myocytes had significantly reduced calcium transient amplitudes ([LCR] 0.142±0.011 vs. [HCR] 0.204±0.016 (360/380 ratio), P<0.05) and slower kinetics of calcium decay compared to HCR myocytes (time from peak to 90% decay: [LCR] 572.7±38.1 vs. [HCR] 458.4±27.6 ms, P<0.05) (Figure 6a and d). Contractile function was also assessed by analysis of sarcomere length kinetics. Compared to myocytes derived from HCR hearts, LCR myocytes had significantly shorter resting sarcomere lengths and slower kinetics of contraction (time to peak: [LCR] 166.0±4.9 vs. [HCR] 141.4±3.3 ms, P<0.05) and relaxation (time from peak to 90% relaxation: [LCR] 426.9±37.7 vs. [HCR] 242.9±18.3 ms, P<0.05) (Figure 6b and c). The sarcomere length shortening amplitude was not different between groups. As discussed in detail below, these results suggest that altered calcium handling may underlie susceptibility to cardiac contractile dysfunction in rats with low aerobic running capacity.

### Analysis of cardiac protein expression in LCR and HCR rats

**Figure 7:**
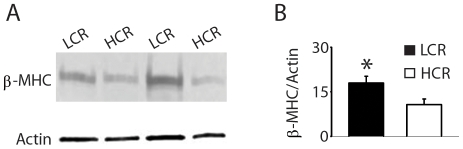
Western blot analysis of β-MHC protein. (a) Representative Western blot of beta myosin heavy chain (β-MHC) protein levels in low and high capacity rats. (b) Summarized mean data showing differences in protein abundance based on densitometric quantitation. Values are expressed as mean±SEM. *P<0.05. n = 6–9/group. LCR, low capacity runner; HCR, high capacity runner.

Based on functional differences in sarcomere shortening and calcium transient kinetics, we sought to determine whether subcellular alterations in protein expression between LCR and HCR rats could explain these findings. Myocardial protein expression analysis showed that LCR rats had significantly greater amounts of β – myosin heavy chain (MHC) compared to HCR rats (Figure 7a and b). Previous studies have shown that the α-MHC isoform (the dominant isoform in rodent hearts) is a fast motor protein compared to the beta isoform [24]. Slight alterations in the stoichiometry of these MHC isoforms can dramatically affect cardiac function [24]. In combination with alterations in calcium handling (Figure 6), the finding that LCR rats had increased expression of β-MHC is consistent with isolated cell data showing slower kinetics of myocyte contractility. β-MHC is a common indicator of upregulation of the fetal gene program associated with heart failure [25]. To address whether this gene program was activated in LCR hearts we analyzed other markers of the fetal gene program (e.g. transcript levels of β-MHC, BNP, and α-skeletal actin analyzed by qRT-PCR). There were no differences in the relative abundance of transcripts for these markers in LCR hearts compared to HCR hearts (Table S1).

Thyroid hormone signaling is also associated with MHC isoform changes [26], [27]. Thus, thyroid hormones were analyzed in LCR and HCR animals to assess whether this variable may be contributing to the observed changes in MHC isoform in LCR rats (Table 4). There were no differences in measures of serum T4, free T4, serum T3, or free T3. However, animals with low intrinsic aerobic capacity had significantly increased levels of thyroid stimulating hormone consistent with a state of pre-clinical hypothyroidism[28] (TSH: LCR, 2.2±0.2 vs. HCR, 0.7±0.2 ulU/mL; P<0.05). Although these data are consistent with a depressed metabolic state in LCR animals stimulating compensatory thyroid production of TSH, these thyroid hormone results indicate that the MHC isoform switch is not the consequence of altered thyroid hormone levels.

**Table 4:** Thyroid Hormone Analysis.

	HCR	LCR
**Serum T4 (ug/dL)**	2.64±0.16	2.92±0.16
**Free T4 (ng/dL)**	1.75±0.04	1.77±0.05
**Serum T3 (ng/dL)**	58.18±2.93	56.74±2.58
**Free T3 (pg/dL)**	2.91±0.07	2.85±0.06
**TSH (ulU/mL)**	0.74±0.16	2.17±0.17*

All values are expressed as mean±s.e.m.

* P<0.05 for LCR vs. HCR.

Terms: T4, Thyroxine; T3, Triiodothyronine. TSH, thyroid stimulating hormone. HCR, high capacity runner (n = 8–32), LCR, low capacity runner (n = 8–32).

Western blot analysis of key calcium handling proteins including the sarco-endoplasmic reticulum ATPase (SERCA2a), phospholamban, the sarcolemal sodium-calcium exchanger, and calsequestrin showed no differences between LCR and HCR rats (Figure S1). To address baseline adrenergic tone that directly affects contractility and calcium handling in myocytes at the molecular level, Western blot detection was performed for tandem serine 23/24 phosphorylation on troponin I as well as serine 16 phosphorylation of phospholamban. This analysis again revealed no difference between LCR and HCR cohorts (Figure S1).

## Discussion

From the cellular to whole organ level, this study provides new insight into the cardiac performance of animals artificially selected for inherent aerobic running capacity. At generation 19 of artificial selection, divergence in intrinsic aerobic capacity as measured by run time to exhaustion was significant, with a 436% difference between high capacity runners (HCR) and low capacity runners (LCR). Analysis of baseline cardiac function by echocardiography and catheter-based conductance micromanometry revealed minor differences in whole animal cardiovascular performance. However, in support of previous evidence showing deficiencies in oxygen transport and oxidative metabolism in LCR rats, our findings reveal significant differences between LCR and HCR rats in cardiac response to a pathophysiological challenge. Animals with low inherent aerobic capacity were highly susceptible to reduced oxygen availability as evidenced by increased susceptibility to hypoxia induced contractile dysfunction compared to animals with high intrinsic aerobic capacity. *In vitro* analysis of isolated myocytes revealed slower kinetics of calcium cycling and contractility. Furthermore, this study provides the first evidence to our knowledge that artificial selection for aerobic capacity (with no inciting injury such as myocardial infarction or pressure overload) results in increased expression of β-MHC protein in animals with intrinsically low aerobic capacity. Together these findings provide new insights into the underlying cardiovascular deficiencies associated with metabolic syndrome.

### Increased β-MHC protein in animals with low intrinsic aerobic capacity

Increased expression of β-MHC in the heart has consistently been reported to occur in the failing heart or in response to various pathophysiological challenges [25], [29], [30]. In this study we showed that β-MHC protein is increased in animals with low intrinsic aerobic capacity. To our knowledge, this is the first study to show that this MHC isoform switching in the heart occurs in response to selection for intrinsic aerobic capacity. Furthermore, no evidence of overt heart failure was seen nor intervention of an inciting stimulus (such as exercise training, myocardial infarction, or pressure overload) was used in this study. This is evidence that the MHC isoform switching occurs as a result of segregation of cardiac risk factors based on artificial selection separating low and high aerobic capacity animals.

The increase in β-MHC protein was not corroborated at the mRNA level based on qRT-PCR analysis. A similar discordance between β-MHC protein and mRNA has been demonstrated to occur in the pathologic heart [25], [30]. For example, Delcayre and colleagues have shown that after seven days of pressure overload induced by aortic constriction, β-MHC protein was increased from 5% to 31% of total MHC without any changes in mRNA. Numerous other studies have shown that, in the absence of changes in mRNA, regulation of MHC protein is highly dependent on a wide range of stimuli [25], [30], [31], [32]. Evidence from this study suggests that a novel stimulus for MHC isoform switching in the adult heart is genetic segregation of factors associated with low intrinsic aerobic capacity.

The implications of this MHC isoform switch reveal fundamental physiological differences between low and high aerobic capacity animals. Specifically, the β-MHC isoform is a slow motor protein that is more economical in its ATPase activity compared to the α-MHC isoform [33]. Prior studies showing a shift in metabolic substrate usage[13] and VO_2max_ measurements [14], [15], [16], [23] between LCR and HCR animals are consistent with a molecular transition to this more economical MHC isoform in rats with intrinsically low aerobic capacity. Despite the energetic benefits, contractile function is markedly compromised when β-MHC is increased in the heart [24], [34]. Furthermore, studies have shown that increased expression of β-MHC in an α-MHC dominant mouse heart causes negative cardiac remodeling in response to numerous stresses (e.g. increases in systolic and diastolic LV dimensions) [35]. These findings are consistent with our observation that LCR hearts have increased geometric dimensions and SV at baseline which may reflect a compensatory remodeling response to this increased β-MHC in the heart. However, the negative inotropic effects of increased β-MHC expression in LCR hearts are not as evident. During the acute hypoxic challenge, we propose that pathologic alterations associated with changes in calcium handling cause the emergence of irregular contractions prior to the manifestation of systolic pump failure attributable to MHC isoform switching.

### LCR hearts have increased susceptibility to irregular pump function during hypoxia

Hemodynamic function of LCR and HCR rats was measured during extended exposure to a controlled state of hypoxia. This acute hypoxic challenge specifically targets the critical aspects of cardiac function regarding oxidative metabolism. The results of this study reveal an increased susceptibility to hypoxia in LCR rats consistent with the finding that risk factors for cardiac disease and, specifically, deficiencies in cardiac calcium homeostasis segregate with aerobic running capacity. Furthermore, this study shows that the primary cause of cardiac failure for LCR rats during exposure to hypoxia was the development of fatal intractable irregular contractions. We hypothesize that the mechanistic basis for this cardiac contractile irregularity during episodes of energetic crisis in LCR rats is multifactorial.

First, based on previous evidence showing differences in metabolic substrate usage between LCR and HCR rats [13], this susceptibility to hypoxia in LCR rats could be due to deficiencies in native oxidative metabolic function. Specifically, previous findings have shown reduced VO_2max_ [14], [15], [16], [23] and shift from lipid metabolism to carbohydrate metabolism[13] in rats selectively bread for low aerobic running capacity. This metabolic shift in LCR hearts would significantly increase the damage caused by fatty acid toxicity during hypoxia. The consequences of this physiological state are poignantly revealed by the development of fatal intractable irregular contractions in LCR rats during episodes of cardiac energetic crisis as seen in this study. Secondly, this study provides mechanistic data supporting a role for MHC isoform switching and altered calcium handling as a basis for the observed susceptibility to pump dysfunction in LCR rats. Overall, the results of this study provide new and important insights into the role of altered calcium homeostasis in the development of cardiac contractile dysfunction during hypoxia and energetic insufficiency.

### A role for altered calcium handling accounting for contractile dysfunction

A role for altered calcium handling has been strongly associated with conduction (e.g. early or delayed after depolarizations) and non-conduction (e.g. calcium induced after contractions) related contractile dysfunction [36], [37], [38]. Thus, decreased calcium cycling coupled with delayed calcium decay during diastole are implicated in cardiac contractile failure [37], [39]. This is consistent with the observations in this study as shown in myocytes isolated from LCR animals. Previously, alterations in calcium cycling have been attributed to proteins critically involved in tightly regulating calcium homeostasis in the myocytes [36]. However, this study revealed no difference in the expression level of these proteins nor in the phosphorylation status of phospholamban, which is involved in regulating calcium cycling through SERCA2a [40].

Previous studies have found that LCR rats have reduced expression of key mitochondrial enzymes required for energy substrate metabolism [12]. In this study it was hypothesized that impairment of mitochondrial function may link reduced fitness to cardiovascular and metabolic disease [12]. In support of this, we speculate that mitochondrial dysfunction and thus decreased availability of ATP that occurs during hypoxia likely exacerbates the dysfunction of SERCA2a-dependent calcium re-sequestration into the SR during diastole in LCR rats. This would explain the observed increase in susceptibility to irregular contractile dysfunction during this challenge.

Studies of calcium cycling implicate calcium leak through the ryanodine receptor (RyR) due to increased RyR open probability (P_o_) as contributing to heart failure-related dysfunction [41], [42]. However, Dibb et al and Venetucci et al report that increased RyR P_o_ in the context of reduced SR calcium is not sufficient to induce sustained arrhythmogenic calcium release events [37], [43]. Only in the context of a correlative increase in pPLN (required to reach some threshold level of SR calcium storage) is increased RyR P_o_ likely to be problematic. The current study shows that LCR rats have significantly lower calcium transients, which suggests that this mechanism is also not contributing to the observed contractile dysfunction in LCR rats during hypoxia. In light of the current data set, we propose that a non-canonical mechanism (perhaps associated with metabolic dysfunction) is implicated in altering the calcium regulation in LCR myocytes thus giving rise to the contractile dysfunction observed in these hearts.

### Conclusion

This study provides insights into the relationship between metabolic syndrome and cardiac function. These data reveal for the first time that selection for low intrinsic aerobic capacity is sufficient to drive changes in MHC isoform composition in the adult heart. Furthermore, low aerobic capacity hearts are highly susceptible to irregular ventricular contractions during an acute hypoxic challenge. Thus, in addition to common therapeutic efforts to ameliorate cardiac energy demands during stress by use of beta-blockers, it has been suggested that immediate intervention at the level of metabolic control (decrease myocardial fatty acid oxidation and increase glucose oxidation) may also be critically important for the stabilization of patients with acute cardiac injury [44], [45], [46]. Lastly, recent studies of LCR and HCR animals has shown that high intensity aerobic interval training (85–90% of maximal oxygen uptake (VO_2max_)) resulted in significant correction of the metabolic syndrome phenotype in LCR animals [47]. In addition to the regulation of metabolic control during acute injury, exercise training is also a clinically relevant mechanism for ameliorating pathologies associated with metabolic syndrome. Recognizing the interaction between gene and environment, this study reveals that polygenic traits associated with risk factors for cardiovascular disease co-segregate with low aerobic fitness.

## Supporting Information

Figure S1 Western blot analysis. (a) Representative Western blots of proteins including serine 23,24 phosphorylation of cardiac troponin I (pTnI), the sarco-endoplasmic reticulum ATPase (SERCA2a), phospholamban (PLN), phospho-phospholamban (pPLN), the sarcollemal sodium-calcium exchanger (NCX), and calsequestrin (CSQ). (b) Summarized mean data for each protein based on densitometric quantitation. Values are expressed as mean±SEM. n = 6–9/group. LCR, low capacity runner; HCR, high capacity runner.
(0.76 MB JPG)

Table S1 All values are expressed as mean±s.e.m. All values are normalized to ribosomal 18s RNA. HCR, high capacity runner; LCR, low capacity runner. N = 16–24/group
(0.01 MB DOC)
