# Supplementary figures and images for: Artificial Selection for Whole Animal Low Intrinsic Aerobic Capacity Co-Segregates with Hypoxia-Induced Cardiac Pump Failure
Source: PLoS One. 2009 Jul 1;4(7):e6117. doi: 10.1371/journal.pone.0006117 (PMC2699480; doi:10.1371/journal.pone.0006117)

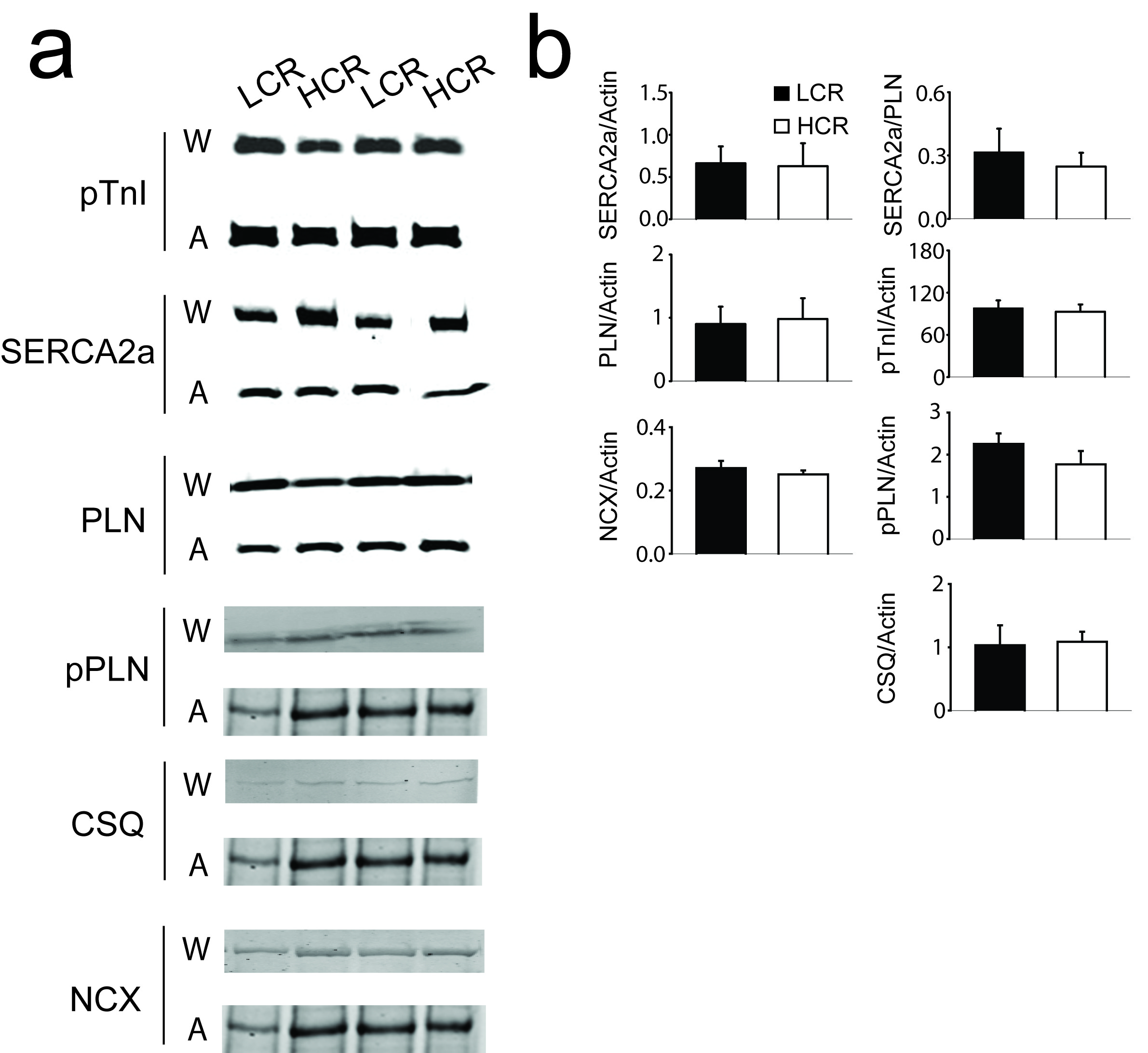

Supplement: Figure S1 — Western blot analysis. (a) Representative Western blots of proteins including serine 23,24 phosphorylation of cardiac troponin I (pTnI), the sarco-endoplasmic reticulum ATPase (SERCA2a), phospholamban (PLN), phospho-phospholamban (pPLN), the sarcollemal sodium-calcium exchanger (NCX), and calsequestrin (CSQ). (b) Summarized mean data for each protein based on densitometric quantitation. Values are expressed as mean±SEM. n = 6–9/group. LCR, low capacity runner; HCR, high capacity runner. (0.76 MB JPG) [file pone.0006117.s001.jpg]
