# Supplementary material for: Artificial Selection for Whole Animal Low Intrinsic Aerobic Capacity Co-Segregates with Hypoxia-Induced Cardiac Pump Failure
Source: PLoS One. 2009 Jul 1;4(7):e6117. doi: 10.1371/journal.pone.0006117 (PMC2699480; doi:10.1371/journal.pone.0006117)
Supplement: Table S1 — All values are expressed as mean±s.e.m. All values are normalized to ribosomal 18s RNA. HCR, high capacity runner; LCR, low capacity runner. N = 16–24/group (0.01 MB DOC) [file pone.0006117.s002.doc]

**Table S1.** Quantitative RT-PCR analysis of fetal gene markers

|  | **HCR** | **LCR** |
| --- | --- | --- |
| BNP | 0.02279 ± 0.01631 | 0.005274 ± 0.001013 |
| α-Skeletal Actin | 0.01415 ± 0.006222 | 0.01476 ± 0.003554 |
| β-MHC | 0.01733 ± 0.007391 | 0.008626 ± 0.001443 |

All values are expressed as mean ± s.e.m. HCR, high capacity runner; LCR, low capacity runner. N = 16-24/group
